# Insertions and deletions trigger adaptive walks in *Drosophila* proteins

**DOI:** 10.1098/rspb.2011.2571

**Published:** 2012-03-28

**Authors:** Evgeny V. Leushkin, Georgii A. Bazykin, Alexey S. Kondrashov

**Affiliations:** 1 Department of Bioengineering and Bioinformatics, Lomonosov Moscow State University, Leninskye Gory 1–73, Moscow 119991, Russia; 2 Institute for Information Transmission Problems of the Russian Academy of Sciences (Kharkevich Institute), Bolshoi Karetny pereulok 19, Moscow 127994, Russia; 3 Life Sciences Institute and Department of Ecology and Evolutionary Biology, University of Michigan, Ann Arbor, MI 48109-2216, USA

**Keywords:** indels, fitness landscape, adaptive walk, McDonald–Kreitman

## Abstract

Maps that relate all possible genotypes or phenotypes to fitness—fitness landscapes—are central to the evolution of life, but remain poorly known. An insertion or a deletion (indel) of one or several amino acids constitutes a substantial leap of a protein within the space of amino acid sequences, and it is unlikely that after such a leap the new sequence corresponds precisely to a fitness peak. Thus, one can expect an indel in the protein-coding sequence that gets fixed in a population to be followed by some number of adaptive amino acid substitutions, which move the new sequence towards a nearby fitness peak. Here, we study substitutions that occur after a frame-preserving indel in evolving proteins of *Drosophila*. An insertion triggers 1.03 ± 0.75 amino acid substitutions within the protein region centred at the site of insertion, and a deletion triggers 4.77 ± 1.03 substitutions within such a region. The difference between these values is probably owing to a higher fraction of effectively neutral insertions. Almost all of the triggered amino acid substitutions can be attributed to positive selection, and most of them occur relatively soon after the triggering indel and take place upstream of its site. A high fraction of substitutions that follow an indel occur at previously conserved sites, suggesting that an indel substantially changes selection that shapes the protein region around it. Thus, an indel is often followed by an adaptive walk of length that is in agreement with the theory of molecular adaptation.

## 1. Introduction

A fitness landscape is a map from the space of all possible genotypes or phenotypes into fitness. Because the key force of evolution, natural selection, appears owing to differences between fitnesses of genotypes, the genotype-to-fitness map is the key determinant of the course of evolution. Indeed, evolution can be thought of as a walk, by an evolving object, on the fitness landscape [1–5]. Adaptive evolution involves climbing up on the fitness landscape, and selectively neutral evolution involves level movements.

**Figure 1.**
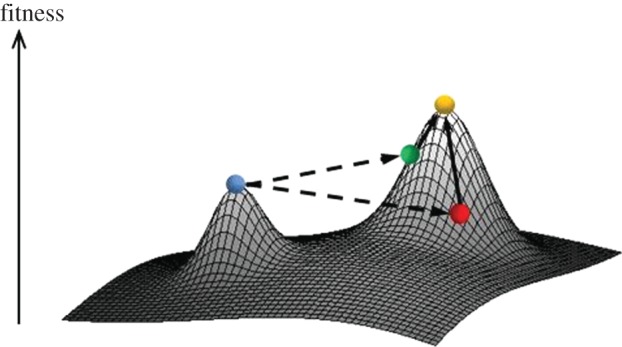
Evolutionary trajectories that involve a major leap in genotype space (see text). The figure shows a generic genotype → fitness map. The original object (blue dot) resides on a local fitness peak. A radical change, such as an insertion or a deletion in a protein sequence, may move the object onto a slope of a higher fitness peak (dashed arrows). This triggers an adaptive walk consisting of a succession of small changes, such as amino acid substitutions (solid arrows), eventually leading to a fitter object.

Figure 1 schematically shows the events caused by a long, instant leap by an evolving object (e.g. an amino acid sequence), within its genotype space [6,7], under the assumption that the object initially resided on a local fitness peak (blue dot). The leap, which may correspond to a fixation of a major mutation in an evolving lineage, can be beneficial (green), neutral or even slightly deleterious (red). In any case, after the leap, the new genotype is unlikely to correspond exactly to a fitness peak. Instead, it is likely to correspond to a slope of a fitness peak that must be higher than the original one, as long as the heights of peaks differ substantially and the leap never involves a major loss of fitness. Thus, we can expect a number of minor adaptive changes to follow the leap, eventually moving the object to the top on the new fitness peak (gold).

On the basis of fairly general considerations, Gillespie concluded that an adaptive walk (i.e. a succession of positive selection-driven allele replacements) in an evolving protein must usually involve two to five replacements [8,9]. This estimate was obtained under the assumption that fitness landscapes are uncorrelated, which certainly does not hold for the fitness landscapes of actual proteins, where similar sequences confer similar fitnesses. In smooth correlated landscapes, longer walks are expected owing to a lower number of suboptimal peaks [10]. On the other hand, numerous studies [11,12] indicate that fitness landscapes of proteins or tRNAs are rugged, owing to the fact that selection is often epistatic, in the sense that relative fitnesses of alleles at a locus depend on the rest of the genome, and therefore allele replacements at different loci depend on each other [13]. The interplay of correlatedness and ruggedness of the fitness landscape makes it hard to predict the length of an adaptive walk *a priori,* and measuring the lengths and durations of such walks in evolving proteins could shed light on the nature of protein fitness landscapes.

An adaptive walk can be triggered by a replacement that affects the direction of selection at a substantial number of loci [1,2]. In the case of evolving proteins, an indel, which usually constitutes a more substantial change to protein structure than a single-nucleotide substitution, may be such a trigger, while subsequent amino acid substitutions may constitute adaptive changes.

## 2. Results

**Figure 2.**
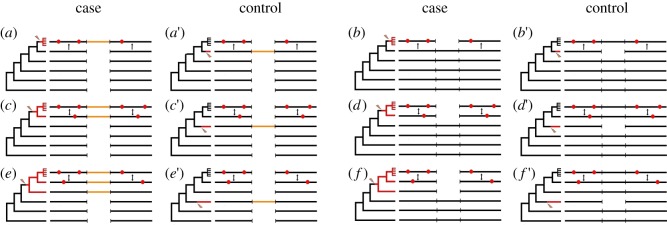
Patterns of insertions or deletions and amino acid substitutions in evolving *Drosophila* proteins used to infer the adaptive walks. On each panel, phylogeny of (((((*D. melanogaster*, *D. sechellia*), *D. erecta*), *D. ananassae*), *D. pseudoobscura*), *D. virilis*) part of the *Drosophila* tree is shown at the left, with the position of an indel marked with a lightning strike, and the post-indel segment of the phylogeny shown in red. For *D. melanogaster*, data on within-species variation, indicated by a comb, were also used. (*a*,*a*′,*c*,*c*′,*e*,*e*′) correspond to insertions, and (*b*,*b*′,*d*,*d*′,*f*,*f*′) correspond to deletions. (*a*–*b*′) (top row) show indels that occurred after *D. melanogaster–D. sechellia* split, (*c*–*d*′) (middle row) show indels that occurred between *D. erecta*–(*D. melanogaster*–*D. sechellia*) and *D. melanogaster*–*D. sechellia* splits, and (*e*–*f*′) (bottom row) show indels that occurred between *D. ananassae*–((*D. melanogaster*, *D. sechellia*), *D. erecta*) and *D. erecta*–(*D. melanogaster*–*D. sechellia*) splits. (*a*–*f*) show indels that occurred in a lineage that eventually led to *D. melanogaster*, and (*a*′–*f*′) show indels that occurred in another lineage. A double-headed arrow indicates the two regions compared for calculating the rate of nucleotide substitutions; a single-headed arrow directed towards a region indicates that for calculating this rate, only the substitutions specific to this region were included. Red dots schematically represent the nucleotide substitutions specific to one of the regions.

We used comparative analysis of orthologous proteins from several species of *Drosophila* to study these hypothetical post-leap adaptive walks (figure 2). Total numbers of indels identified are shown in table 1. We applied the McDonald–Kreitman (MK) test [14,15] to amino acid substitutions that occurred, within 100 amino acids of the site of an indel, in the *Drosophila melanogaster* lineage after its split from the *Drosophila sechellia* lineage, by relating data on *D. melanogaster*—*D. sechellia* divergence to data on polymorphism within *D. melanogaster* at synonymous and non-synonymous sites (electronic supplementary material, figures S1–S4). We contrasted the results of this test for cases when an indel occurred in the *D. melanogaster* lineage (‘case’ sample) with the corresponding cases when an indel occurred in a sister lineage (‘control’ sample; figure 2*a* versus *a*′, *b* versus *b*′, etc.). Obviously, in the ‘case’, but not in the ‘control’, substitutions that occurred recently in the  *D. melanogaster* lineage could possibly be triggered by an indel.

**Table 1.** Length distribution of non-frameshifting indels in *Drosophila* coding regions. Each row corresponds to phylogenetic configuration in figure 2. *a*, *a*′, *c*, *c*′, *e*, *e*′ are insertions, and *b*, *b*′, *d*, *d*′, *f*, *f*′ are deletions. *a–b*′ are indels that occurred after the *D. melanogaster–D. sechellia* split, *c–d*′ are indels that occurred between *D. erecta*–(*D. melanogaster*–*D. sechellia*) and *D. melanogaster*–*D. sechellia* splits, and *e–f*′ are indels that occurred between *D. ananassae*–((*D. melanogaster*, *D. sechellia*), *D. erecta*) and *D. erecta*–(*D. melanogaster*–*D. sechellia*) splits. *a*, *b*, *c*, *d*, *e*, *f* are indels that occurred in a clade that includes *D. melanogaster*, and *a*′, *b*′, *c*′, *d*′, *e*′, *f*′ are indels that occurred in a clade that does not include *D. melanogaster*.

phylogenetic configuration from figure 2	indel length (codons)				
	1	2	3	4	5+
*a*	104	29	11	3	7
*a′*	145	54	18	16	23
*b*	59	27	16	8	5
*b′*	205	86	45	25	43
*c*	120	20	1	3	1
*c′*	195	148	53	37	55
*d*	68	32	18	14	4
*d′*	269	67	46	21	44
*e*	519	124	31	36	16
*e′*	2719	1064	434	256	468
*f*	515	176	74	41	50
*f′*	1747	646	326	195	196

**Figure 3.**
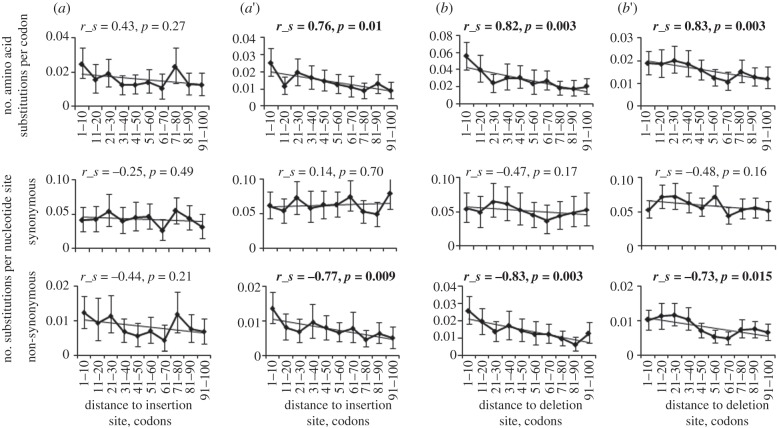
Increased rate of non-synonymous, but not synonymous, nucleotide substitutions close to an indel site. (*a*–*b*′) of this figure correspond to (*a*–*b*′) of figure 2. The top row shows the number of amino acid substitutions per amino acid site, the middle row shows the number of synonymous substitutions per synonymous nucleotide site and the lower row shows the number of non-synonymous substitutions per non-synonymous nucleotide site, at different distances from the site of the indel. Error bars are 95% CIs based on 1000 bootstrap trials. Correlation between the distance and the number of substitutions was tested using Spearman's test; bold indicates significant (*p* < 0.05) correlations.

**Figure 4.**
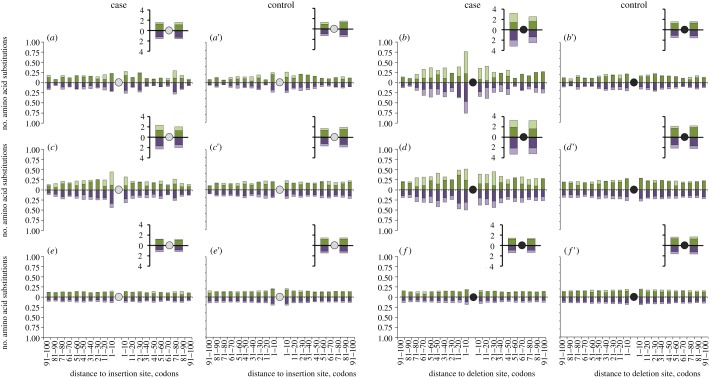
Excess of adaptive amino acid substitutions around the recent indels in the *D. melanogaster* lineage. Panels correspond to analyses shown in figure 2. The grey (black) circles identify the insertion (deletion) site, and the bars to the left (right) of the circles show substitutions at different distances from it towards the N-end (C-end). Heights of the bars above and below each horizontal axis show the total numbers of amino acid substitutions that occurred at the terminal segment of the *D. melanogaster* lineage (after its divergence from *D. sechellia*) in this protein region. Above each horizontal axis, the fraction of these substitutions that were driven by positive selection, estimated by the McDonald–Kreitman test, is shown in light green, and the remaining fraction is shown in dark green. Below each horizontal axis, the fraction of substitutions that occurred at conservative sites that are invariant in the six basal *Drosophila* species not affected by an indel (*D. pseudoobscura*, *D. persimilis*, *D. willistoni*, *D. virilis*, *D. mojavensis* and *D. grimshawi*) is shown in light purple, and the remaining fraction is shown in dark purple. The insets show the corresponding values summed over all distances between 1 and 100 codons from the indel site.

Figures 3 and 4 present data on evolution and positive selection in the terminal segment of the *D. melanogaster* lineage (after *D. sechellia* branching off) at sites adjacent to the site of an indel. Consistent with earlier observations [16,17], we see that indels tend to occur within rapidly evolving regions of proteins, as evidenced by higher rates of amino acid substitutions within several tens of amino acids from the sites of indels, compared with regions more remote from indel sites, both when an indel occurred in the *D. melanogaster* lineage and when it occurred in a sister lineage (figure 3, top row). However, separate analysis of synonymous and non-synonymous nucleotide sites shows that this pattern is not due to increased mutation rate close to indel sites, as was proposed by McDonald *et al*. [18]. Indeed, only the non-synonymous substitutions, not the synonymous substitutions, are significantly more frequent near the indel sites (figure 3, middle versus bottom row; electronic supplementary material, figures S1 and S2).

Accelerated evolution associated with an indel event is also not owing to increased mutation rate caused by a heterozygous indel [16], for the same reason, and also because the frequencies of synonymous substitutions and synonymous polymorphisms do not depend on whether an indel occurred in the *D. melanogaster* lineage or in a sister lineage (electronic supplementary material, figures S1 and S3). Finally, the frequency of non-synonymous polymorphism also remains unchanged after an indel event (electronic supplementary material, figure S4), implying that acceleration is not owing to reduction in selective constraint, as suggested by Zhang *et al*. [17]. In contrast, the rate of non-synonymous nucleotide substitutions was significantly elevated in regions of several tens of codons from an indel site when the indel occurred in the *D. melanogaster* lineage, compared with when it occurred in a sister lineage (electronic supplementary material, figure S2). The elevation was pronounced for insertions corresponding to the phylogenetic configuration in figure 2*c*, and for deletions corresponding to the phylogenetic configuration in figure 2*b,d* (electronic supplementary material, figure S2). On average, after an insertion, 1.03 ± 0.75 excess amino acid substitutions occurred (figure 4*a* versus *a*′: 0.33, *c* versus *c*′: 0.71), and after a deletion, 4.77 ± 1.03 excess amino acid substitutions occurred (figure 4*b* versus *b*′: 2.60, *d* versus *d*′: 2.17) within the protein segment of 200 amino acids centred at the site of the indel (see §4 for details).

The fact that the difference in rates is limited to non-synonymous substitutions implies that this significant (figure 3; electronic supplementary material, figure S5) excess of amino acid substitutions is entirely owing to positive selection. Formally, the fraction of adaptive amino acid substitutions can be calculated using the MK test [14,15]. As shown in figure 4, the vast majority of amino acid substitutions triggered by the indel event in the vicinity of the indel site was driven to fixation by positive selection. Indeed, the somewhat increased rate of amino acid substitutions around the site of the indel when the indel occurred outside the *D. melanogaster* lineage (electronic supplementary material, figure S5) was matched by increased non-synonymous polymorphism (electronic supplementary material, figure S4), and therefore no increase in the fraction of adaptive amino acid substitutions was observed, compared with the protein segments further away from the indel site (figure 4*a*′,*b*′, etc.). By contrast, the excess amino acid substitutions that occurred after the indel in  *D. melanogaster* lineage were adaptive, as they could be entirely explained by positive selection (figure 4*b–d*). For unknown reasons, most of such substitutions occurred upstream of the indel site (figure 4*b*–*d*; electronic supplementary material, figure S5). The difference between the closest 10 amino acids upstream of the indel site and 10 amino acids downstream of it was most pronounced for figure 4*b* (Fisher's exact test; *or* = 2.29, *p* = 0.0006), and less significant for figure 4*c* (Fisher's exact test; *or* = 1.41, *p* *=* 0.057) and figure 4*d* (Fisher's exact test; *or* = 1.35, *p* = 0.060).

The observed increase in the rate of adaptive evolution depends on the time since the indel. Although we only consider substitutions within the terminal segment of the *D. melanogaster* lineage, we were able to compare indels of different ages. More ancient indels (i.e. those that occurred between branchings off of *Drosophila ananassae* and *Drosophila erecta*; figure 2*e*–*f*′) did not increase the number of amino acid substitutions in the terminal segment of the *D. melanogaster* lineage (figure 4*e*–*f*′), indicating that a post-indel adaptive walk does not take a very long time.

A strong contrast between insertions and deletions was observed. Compared with an insertion, a deletion triggers substitutions in a much wider region of a protein (up to approx. 100 amino acids, compared with approx. 40 amino acids for insertion), and a larger fraction of the substitutions within this entire region are positively selected (figure 4*a* versus *b*, *c* versus *d*). For deletions (figure 4*b*–*b*′), but not for insertions (figure 4*a*–*a*′), a significant increase in the rate of adaptive substitutions was observed even when the indel occurred in the terminal segment of the *D. melanogaster* lineage, where a weaker effect can be expected because substitutions that occurred after the indel could not be distinguished from those that occurred before it.

**Figure 5.**
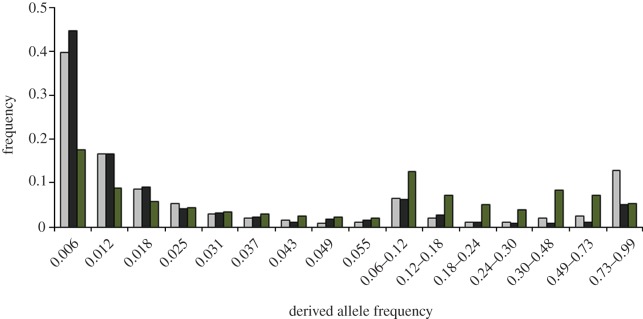
Distribution of allele frequencies for polymorphic insertions and deletions of single amino acids in *D. melanogaster* proteins. Mean frequencies of a derived indel are 0.097 for insertions (grey bars) and 0.057 for deletions (black bars); both values are well below 0.175, the mean allele frequency predicted by the infinite sites model under neutrality (green bars) [19].

The contrast between insertions and deletions may be due to a stronger impact of a deletion on protein structure and function. Data on frequencies of insertions and deletions segregating within the *D. melanogaster* population (figure 5) indicate that deletions segregate at lower frequencies compared with insertions, and therefore are consistent with this explanation. The value of Tajima's D [20] is lower for deletions (−1.64) than for insertions (−1.19), indicating that an average deletion is less neutral than an average insertion.

Among post-indel substitutions, a higher fraction occurred at sites that were conserved among more distant *Drosophila* species, compared with the substitutions that did not follow an indel (figure 4, light purple versus dark purple bars). Again, this effect was more pronounced for deletions (Fisher's exact test; figure 4*b* versus *b*′: *or* = 4.61, *p* ≈ 0; figure 4*d* versus *d*′: *or* = 2.19, *p* ≈ 0; figure 4*f* versus *f*′: *or* = 1.61, *p* ≈ 0) than for insertions (Fisher's exact test; figure 4*a* versus *a*′: *or* = 0.88, *p* = 0.66; figure 4*c* versus *c*′: *or* = 1.36, *p* = 0.006; figure 4*e* versus *e*′: *or* = 1.07, *p* = 0.24). Therefore, an indel apparently changes selection acting at individual amino acid sites around it, and thus constitutes a long leap on the protein fitness landscape (figure 1).

## 3. Discussion

Our analysis confirms the observation [17] that an indel in an evolving protein is followed by accelerated evolution around it. Single-nucleotide and indel mutation rates are correlated in the genome [16,18,21]; in addition, single-nucleotide and indel polymorphisms and replacements are further correlated owing to their tendency to occur in regions of reduced selection [17]. However, in our analysis, we control for both these factors, and still observe a significant increase in the rate of evolution after an indel in its vicinity. This increase is limited to amino acid-changing mutations, and is caused by positive selection. Positive selection that follows an insertion or, in particular, a deletion often operates at sites that were previously conservative, and thus were not subject to positive selection before the triggering indel.

Accelerated adaptation after a radical mutation is consistent with pervasive epistatic interactions in a protein, and is probably associated with adjustment of the protein sequence to a new, indel-modified protein structure. Positive selection-driven amino acid replacements in the vicinity of an earlier amino acid replacement were previously observed in evolution of mammalian [22] (electronic supplementary material, figure S1) and drosophilid [23] proteins; this effect spanned a region of at least 20 codons, and was stronger when the second substitution compensated the charge change introduced by the first substitution [23]. A radical mutation such as an indel or a charge-changing substitution therefore corresponds to a leap on the fitness landscape, and accelerated evolution subsequent to it corresponds to an adaptive walk triggered by this mutation.

The fact that an average indel triggers an adaptive walk indicates that it substantially changes the fitness landscape for the surrounding amino acids. The length of the walk is approximately 1 for insertions and approximately 5 for deletions. This difference is apparently owing to the differences in the effect of insertions and deletions on the protein structure: an average fixed deletion affects it more [17], as indicated also by stronger selection against deletions (figure 5). Therefore, a deletion is associated with a longer leap on the fitness landscape. As correlations in the fitness landscape are reduced with the length of the leap [1], the height of the new peak can be expected to be less similar to the height of the old peak after a deletion; and as the new peak is unlikely to be much lower, a greater difference is expected between their heights when they are further away from each other. Importantly, an indel leads to adaptive evolution even of previously conserved amino acids, further supporting the suggestion that it may radically affect the fitness landscape; this effect is again more pronounced for deletions, consistent with their stronger effect on the protein structure. Together, our results indicate that post-indel adaptive walks have a considerable role in adaptation.

The fact that an indel that has occurred in *D. melanogaster* lineage between the *D. erecta* and *D. sechellia* splits affects the evolution after the *D. sechellia* split (figure 4*c*–*d*′) indicates that the adaptive walk is still not over after approximately 0.1 silent substitutions per site, and thus takes a considerable time after an indel event. This time lag could be due to long waiting times for individual mutations, and/or due to the fact, that to be adaptive, mutations may have to occur in a specific order [12]. Still, the adaptive walk is confined to the time intervals during which less than one neutral substitution occurs, on average, per nucleotide site, indicating that the positive selection involved has a substantial strength (an increase in the rate of adaptive evolution is observed in figure 4*c*–*d*′, but not in *e*–*f*′).

Even after an adaptive walk is over, and the overall rate of evolution returns to the background level, the disproportional number of substitutions continues to occur at previously conservative sites. This indicates that the indel-caused change in the conservation of individual amino acid sites is permanent, probably owing to restructuring of the protein (figure 4*e*–*f*′).

Indel-triggered adaptive walks in evolving proteins show that the ruggedness of their fitness landscapes plays a substantial role in their long-term evolution. More detailed studies of the key properties of fitness landscapes, such as ruggedness, correlatedness and the variation of the relative heights of peaks, both in the natural and in the experimental systems, will elucidate this role further. Specifically, the entire distribution of the lengths of adaptive walks can be obtainable experimentally. Studies of long-term evolution may be informative of the properties of the adaptive walks that are actually realized in nature, and data on the lengths of such walks could shed light on the rate of protein adaptation.

## 4. Material and methods

### (a) Genome sequences data

Full genome alignments of 11 *Drosophila* species to *D. melanogaster* (dm3, BDGP release 5) were downloaded from the University of California, Santa Cruz database [24] (http://hgdownload.cse.ucsc.edu/goldenPath/dm3/multiz15way/). Coding sequences were extracted from the alignments according to FlyBase annotation of canonical splice variants in *D. melanogaster*. SNP data for *D. melanogaster* were obtained from complete genotypes of 162 inbred lines downloaded from http://www.hgsc.bcm.tmc.edu/projects/dgrp/freeze1_July_2010/sequences.

### (b) Identification of insertions and deletions

Reference sequences of six species of *Drosophila,* namely *D. melanogaster, D. sechellia, D. erecta, D. ananassae, D. pseudoobscura* and *D. virilis,* were used to identify the sites of indels. Only the indels within protein-coding regions with lengths in multiple of three nucleotides (i.e. not giving rise to frameshifts) were analysed (when an indel spanned the border of the exon, only the exonic part of the indel was counted). Indels were polarized using the sequences of *D. pseudoobscura* and *D. virilis* as shown in figure 2; indels not conforming to the depicted phylogenies (e.g. multiple coincident or overlapping indels in different clades) were excluded from the analysis. Additionally, to avoid regions of poor alignment, we required that none of the six analysed sequences carried any gaps or other non-ATCG characters in the 10 bp flanking the indel from the left and 10 bp flanking it from the right (using somewhat shorter and longer window lengths led to similar results). The numbers of indels left after this filtering are shown in table 1.

### (c) Polymorphic indels calling in *Drosophila melanogaster*

The calling procedure for polymorphic indels was performed with mpileup from SAMtools package [25] (v. 0.1.17, http://samtools.sourceforge.net). We performed calling on sequencing data obtained from http://www.hgsc.bcm.tmc.edu/projects/dgrp/freeze1_July_2010/Illumina [26]. Calls were filtered according to the Phred quality score; only the calls with the PhredScore greater than 10 were retained. Alignment segments containing the indels polymorphic in *Drosophila melanogaster* were realigned by MUSCLE (v. 3.7) to increase the quality of alignment with other insect species. *Drosophila sechellia* and *D. erecta* were used to polarize the indels; indels were discarded when these two outgroups disagreed, or if more than 50 per cent of *D. melanogaster* individuals had no data for this region of the genome. The fraction of sequencing and assembly errors should be the highest among the frameshifting indels, because such indels can be expected to be rare. Therefore, to estimate the upper threshold of the error frequency, we used the ratio of the number of frameshifting indels in the protein-coding regions to the number of indels of lengths not a multiple of three in short introns. This ratio was approximately 0.05 for insertions and deletions with frequencies below 15 per cent, and it was only 0.007 for insertions and deletions with frequencies above 15 per cent, implying that the fraction of erroneous indel calls is very low.

### (d) McDonald–Kreitman test

Codons at different distances from the site of the insertion were used for the MK test. We only included a nucleotide site in the test if, in each of the six analysed species, it was flanked by 10 non-gapped, ATCG-only nucleotides to the left and 10 such nucleotides to the right (the only gaps allowed were those associated with the focal indel). Only non-degenerate (four-fold degenerate) nucleotide sites were considered non-synonymous (synonymous), meaning those sites in which any substitution led (did not lead) to an amino acid substitution. Synonymous divergence *D*_s_, non-synonymous divergence *D*_n_, synonymous polymorphism *P*_s_ and non-synonymous polymorphism *P*_n_ were assessed as the fraction of mismatches at corresponding sites. For indels that occurred at the terminal segment of *D. melanogaster* lineage (figure 2*a*–*b*′), only the divergence along a fraction of the *D. melanogaster* lineage could be affected by the indel. Therefore, we counted *D*_s_ and *D*_n_ only for the substitutions along the *D. melanogaster* lineage by including only the nucleotide sites matching between *D. sechellia* and *D. erecta*. For indels that occurred at earlier segments of *D. melanogaster* lineage (figure 2*c*–*f*′), the divergence along both the *D. melanogaster* and *D. sechellia* lineages was affected by the indel. Therefore, we counted *D*_s_ and *D*_n_ for all sites, no matter in which of the two lineages the substitutions occurred.

The proportion of non-synonymous nucleotide substitution driven by positive selection was estimated (from data in the electronic supplementary material, figures S1–S4, tables S1–S4) as *α* = 1 – (*P*_n_/*P*_s_)/(*D*_n_/*D*_s_) [15]*. *α** can be biased by weakly deleterious mutations segregating within a population, and the recommended remedy is exclusion of low-frequency alleles [27]. Therefore, we considered a site polymorphic only when each allele was present in at least two individuals.

### (e) Length of adaptive walks

The length of an adaptive walk was defined as the number of amino acid substitutions in the terminal segment of the *D. melanogaster* lineage triggered by an indel, calculated as follows. For indels that occurred at the terminal segment of *D. melanogaster* (figure 2*a*,*b*) or *D. sechellia* (figure 2*a*′,*b*′) lineage, we counted the amino acid differences between *D. melanogaster* and *D. sechellia* at amino acid sites matching between *D. sechellia* and *D. erecta,* thus including only *D. melanogaster*-specific replacements. For indels that occurred at earlier segments of *D. melanogaster* lineage (figure 2*c*–*f*), or at terminal lineages of *D. erecta* (figure 2*c*′,*d*′) or *D. ananassae* (figure 2*e*′,*f*′), and which therefore affected (or did not affect) *D. melanogaster* and *D. sechellia* equally, we counted the total numbers of amino acid differences between *D. melanogaster* and *D. sechellia*, and divided it by two. Finally, the length of the adaptive walk was calculated as the difference between the number of substitutions that occurred in the terminal segment of *D. melanogaster* lineage when the indel also occurred in the *D. melanogaster* lineage, and this number when the indel occurred in the sister lineage (figure 4*a* versus *a*′ and *c* versus *c*′ for insertions; figure 4*b* versus *b*′ and *d* versus *d*′ for deletions). More ancient indels that occurred before *D. erecta* split were not considered, since they triggered no adaptive substitutions in the terminal segment of the *D. melanogaster* lineage (figure 4*e* versus *e*′, *f* versus *f*′). The lengths of the adaptive walks were obtained separately for each bin of 10 amino acids from the indel site (figure 4); the final value was obtained as the sum over all 20 such bins (10 to the left and 10 to the right).

95% CIs for all estimators were determined by randomly bootstrapping the regions around the indels with replacement (electronic supplementary material, figures S1–S5 and tables S1–S5). 1000 bootstrap trials were used.

### (f) Evolution at amino acid sites of different conservatism

The amino acid sites were considered conservative if the encoded amino acid was invariant between *Drosophila pseudoobscura*, *Drosophila persimilis*, *Drosophila willistoni*, *Drosophila virilis*, *Drosophila mojavensis* and *Drosophila grimshawi*. All remaining sites were considered non-conservative. The fraction of the conservative sites in the region around the indel was similar when the indel occurred in the *D. melanogaster* lineage and in the sister lineage.

### (g) Theoretical distribution of allele frequencies

The expected frequency distribution for a neutral allele was derived from the expected relative times for which the frequency of the derived allele resides within a given interval as *f*(*x*) = 1/*x,* where *x* is the frequency of the derived allele [19,28].
